# Is There Extra Cost of Institutional Care for MS Patients?

**DOI:** 10.1155/2013/713627

**Published:** 2013-09-14

**Authors:** Katia Noyes, Alina Bajorska, Bianca Weinstock-Guttman, Dana B. Mukamel

**Affiliations:** ^1^University of Rochester, USA; ^2^State University of New York at Buffalo, USA; ^3^University of California, Irvine, USA

## Abstract

Throughout life, patients with multiple sclerosis (MS) require increasing levels of support, rehabilitative services, and eventual skilled nursing facility (SNF) care. There are concerns that access to SNF care for MS patients is limited because of perceived higher costs of their care. This study compares costs of caring for an MS patient versus those of a typical SNF patient. We merged SNF cost report data with the 2001–2006 Nursing Home Minimum Data Set (MDS) to calculate percentage of MS residents-days and facility case-mix indices (CMIs). We estimated the average facility daily cost using hybrid cost functions, adjusted for facility ownership, average facility wages, CMI-adjusted number of SNF days, and percentage of MS residents-days. We describe specific characteristics of SNF with high and low MS volumes and examine any sources of variation in cost. MS patients were no longer more costly than typical SNF patients. A greater proportion of MS patients had no significant effect on facility daily costs (*P* = 0.26). MS patients were more likely to receive care in government-owned facilities (OR = 1.904) located in the Western (OR = 2.133) and Midwestern (OR = 1.3) parts of the USA (*P* < 0.05). Cost of SNF care is not a likely explanation for the perceived access barriers that MS patients face.

## 1. Introduction

Multiple sclerosis (MS) affects younger adults and leads to permanent disability. As a result of MS progression and relapses, many MS patients require increasing levels of medical and supportive services and eventually may need long-term skilled nursing facility (SNF) care.

Since 1999, the United States Supreme Court's decision of *Olmstead v. L.C.*, a ruling that requires states to eliminate unnecessary segregation of persons with disabilities and to ensure that persons with disabilities receive services in the most integrated setting appropriate to their needs, has shifted the landscape of services for individuals with disability [1]. Earlier reports by MS patient organizations, care-givers groups, and policy advocates expressed concerns that SNFs and long-term care insurance plans may perceive cost of care for an individual with MS higher than the costs of caring for a typical (elderly and frail) SNF resident, and, hence, they may selectively encourage MS patients with skilled nursing care needs to leave their facility or deny admission [2–4]. Research suggests several potential reasons for this “cream-skimming” behavior that may lead to difficulties for MS patients getting the skilled nursing care they need. These factors include younger age of MS patients compared with other facility residents that may substantially reduce MS patient satisfaction with care and quality of life, specific symptoms, and healthcare problems associated with MS (i.e., pain, cognitive impairment, numbness, spasticity, and fatigue) and types of services they may require (i.e., rehabilitating and behavioral or occupational therapy) [5–7]. 

In this study, we aimed to examine the incremental costs of care for SNF residents with MS and to answer the question whether an MS resident in a nursing home imposes additional costs on the facility, compared with the typical, or average, nursing home resident. We tested two distinct hypotheses. First, we examined whether SNFs with a higher number of MS residents-days are different from SNFs with fewer MS residents. Second, using a cost function model, we calculated the incremental costs due to an increase of 1 percent in MS residents-days provided by a facility. 

## 2. Methods

### 2.1. Theoretical Background: Cost Function Estimation

Following the methods used in our previous work [8], we estimated facility cost functions that explicitly modeled the relationship between the costs of care provided by the nursing home, the costs of inputs used to produce the care (e.g., wages of nurses aides), and the amount of care provided (e.g., number of nursing home days). In order to be able to assess the incremental costs due to caring for MS residents, we included in the model *the percent of MS inpatient days* in the facility. The estimated coefficients from this model allowed us to calculate the incremental costs (actually, log(costs)) faced by a provider treating an additional 1% of MS patients. We note that because the model included case-mix-adjusted days and admissions, the differential cost estimated for MS patients was the cost *above and beyond* any cost differences due to differences in health statuses and demographics (i.e., age and gender) between MS and non-MS residents.

We estimated the total cost using a hybrid cost function, following Grannemann and Brown [9] and Nyman [10] to allow flexibility in the functional form. We estimated the model of the following general form:

(1) log⁡⁡C=ϕX+∑iailog⁡⁡Wi+βCMO+γ%MS,

where *C* is the annual total cost of the provider, *X* is a set of potential variables likely to influence costs (e.g., profit versus nonprofit ownerships), *W* are the county-year means of average facility wage, CMO is the case-mix-adjusted measure of outputs such as the Resource Utilization Groups (RUGs) score-adjusted nursing home days and admissions [11, 12], and %MS is the percentage of days provided to MS patients. Nursing home case-mix indices (CMIs) are used by the CMS to adjust facility reimbursement based on the severity of resident illness in each facility in a given time. The CMIs at admission and annual averages were calculated using the methodology developed by the CMS [11, 12]. Since there were multiple records per facility, we adjusted for the correlation among observations provided by the same facility (see the appendix for more details).

### 2.2. Data Sources

This study was based on the analysis of two databases: the 2001–2006 skilled nursing facility (SNF) cost reports for all USA Medicare-certified facilities and the national Minimum Data Set (MDS) data. The SNF Medicare cost reports are prepared for fiscal purposes by all Medicare-certified free-standing SNFs and contain information about facility characteristics, facility wages, cost allocation based on cost center, and services provided [13]. The Minimum Data Set (MDS) [6, 14] is a standardized screening and assessment tool which forms the foundation of the comprehensive assessment for all residents (regardless of the source of payment) of Medicare- and Medicaid-certified nursing homes. Together, these two data sources provided us with the necessary information to develop cost function and assess marginal cost of providing care to SNF residents with MS compared with costs for residents without MS.

### 2.3. Analytical Datasets

**Figure 1:**
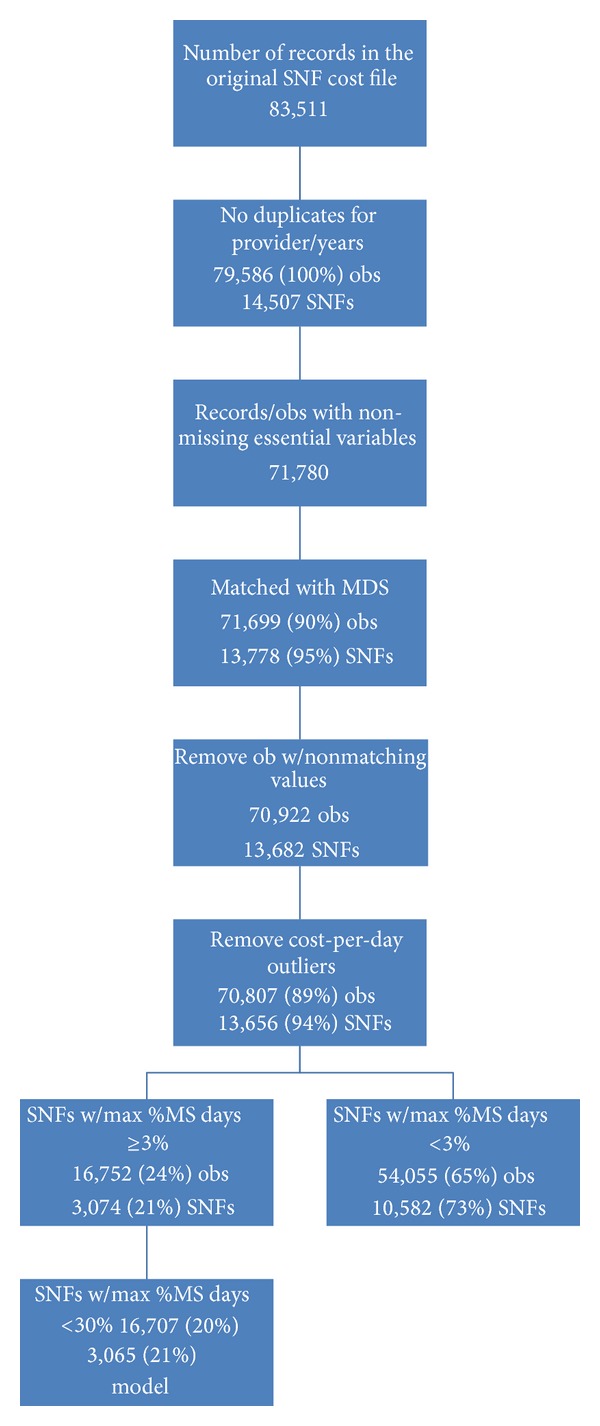
Medicare cost reports: flowchart. The hybrid cost function was estimated on a subset of SNFs including about 20% of facilities with more than 3% of annual residents-days attributed to patients with MS. The dependent variable was defined as logarithmic transformation of *skilled nursing facility inpatient costs*. The independent variables included facility percent of MS days, county-year mean of facility average wage, number of inpatient days and admissions, facility for-profit and ownership status, area competition index, and interactions between them. MS = multiple sclerosis; SNF = skilled nursing facility.

We merged the SNF cost report data with the MDS data using facility Medicare numbers and MDS facility internal IDs (Figure 1). MS residents were identified based on the MS status indicator reported by the admitting physician in the MDS dataset. We used the MDS health status assessment data to calculate percentage of MS days (i.e., inpatient days of care of MS patients as a fraction of total facility inpatient days) and facility case-mix indices (CMIs) [15]. The final dataset without duplicates included 13,656 nursing homes (94% of all SNFs represented in the 2006 cost reports) (Figure 1). 

We assumed that, for facilities with very low proportion of MS days (<3%), the cost of care for MS patients is unlikely to have any impact on facility financial stability. Hence, for our analysis, we identified a *subset of SNFs* with an adequate volume (number of MS patients) for financial impact statistical assessment (facilities with a percentage of MS days in a facility as ≥3%, which is the 90th percentile in the distribution of SNF mean percentage of MS days, or about 20% of all SNFs) during at least one year. Forty-four (44) records for 9 facilities were excluded as outliers: 3 facilities that specialize in care for MS patients (more than 30% of MS days) and 6 facilities based on poor MIXED model fit using influence diagnostics [16]. The final analytical dataset for the cost function model included 16,707 observations from 3,065 facilities.

### 2.4. Analysis

To test our hypotheses, we performed the analyses in two stages. First, we examined how facilities that serve MS patients are different from facilities that serve none or a few (Tables 1(a) and 1(b)). Then, we focused on the facilities that serve a substantial number of MS patients (defined as at least 2.8% of their patients having a diagnosis of MS). For this subset of SNFs, we estimated the impact of the percentage of MS days on facility costs (Table 2) and sought out any evidence whether facilities with high-percentage MS patients may be a substantively different class of SNFs (Table 3).

#### 2.4.1. Characterizing Facilities by Proportion of Admitted MS Patients

We developed a logistic model to compare facilities that consistently admit a greater number of MS patients (higher MS patient volume) with facilities that only treat a few or no MS patients. The binary dependent variable was whether this facility has a high MS volume (results reported in Table 1(b) are for facilities with more than 3% of MS inpatient days). Independent variables included the following predictors characterizing SNFs: US region of SNF location, type of facility (not for profit, for profit, or governmental), facility mean cost per day, annual number of inpatient days and admissions, and facility average case-mix index. The facility mean cost per day was adjusted for case-mix, inflation, and geographical variation in medical care prices (CMS 2010 Wage Index for skilled nursing facilities). This facility-level analysis was performed on 13,656 SNFs. 

We repeated the logistic and mixed regression model analyses while varying our definition of “high MS patient volume” facility (using 1%, 1.5%, 2%,…, 6% thresholds). The findings were consistent for all threshold values, and, hence, only results with 3% cutoff are presented in Table 1(b).

#### 2.4.2. Estimating Average Facility Daily Costs Using Cost Function

For the subset of SNFs with a percentage of MS days in a facility as ≥3%, we developed a cost function to estimate average facility costs. The dependent variable was defined as the logarithmic transformation of *skilled nursing facility inpatient costs.* To calculate inpatient costs, we used inpatient-to-total revenue ratio from the SNF cost report. 

The unit of analysis for the cost function model is facility-fiscal year, with maximum of six records (i.e., years of data) per facility. The independent variables included facility percentage of MS days (average by facility and by facility-year), county-year mean of facility average wage, and number of inpatient days and admissions, as well as the facility for-profit and ownership status, the area competition index, and the interactions between them. The logarithmic transformations were also used for the following independent variables in the model: total number of inpatient days, total number of admissions, and wages. The wages were calculated as year-specific county means of average nursing home staff wages [13]. For the counties in rural areas that had only one facility per county, we used state/rural/year averages instead. Number of inpatient days was adjusted for severity of facility population illness using facility CMI [11]. We also included competition variable (1-HHI), where HHI is the Herfindahl-Hirschman index [17, 18], and type of facility ownership (i.e., government owned, not for profit, or for profit). 

We inflated costs and wages to 2006 level, the last year of the dataset, using the Medical Care component of the Consumer Price Index [19]. Since the reported periods were defined as facility-fiscal year which varied by nursing homes, we calculated separate inflation factors for each time period. 

In this model, we controlled for facility effect by using facility-level random effects and by specifying the covariance of residuals as autoregressive order 1 (AR1) (the appendix).

## 3. Results

### 3.1. Facility Characteristics and Subgroups

On average, 1.2% of annual facility days were attributed to MS patients. We found that facilities that provided care to a larger percentage of MS patients were much more likely to be located in the West (28% versus 14%, *P* < 0.001) and the Midwest (41% versus 30%) of the country and much less likely to be in the South (13% versus 37%, Table 1). Facilities with high percentage of MS patients also had fewer admissions, even after controlling for the number of care days (potentially, indicating lower patient turn-around and more custodial or permanent stays) (OR = 0.988; *P* < 0.001) and were more likely to be government-owned (OR = 1.904; *P* < 0.001) (Table 2). Of note, facilities with high percentage of MS patients, above 35, did not have a higher average case-mix index nor did they have higher costs per day (Table 2).

We also examined factors that explain variation in the percentage of MS-specific care days for those SNFs that care for a substantial number of MS residents—in the top 20th percentile of the distribution—that is, with a mean percentage of MS days as >3% (see Table 3). Besides some regional variation, the only other statistically significant difference was that lower facility mean CMI was associated with higher percentage of MS days (coefficient −1.733, *P* = 0.011) (Table 3). This translates into a 0.27 difference in mean facility CMI (equal to the difference between the 95th and 5th percentiles) being equivalent to 0.5% decrease in the facility percentage of MS care days.

### 3.2. Impact of Percentage of MS Patients in Facility on Facility Costs

Table 4 presents the estimated cost function. The estimates show the expected behavior of a cost function with costs increasing with admissions, days, case mixes, and wages. However, after controlling for facility characteristics, we found no significant effect of the percentage of MS days on the facility inpatient costs (*P* = 0.26) suggesting that MS patients do not impose higher costs above and beyond those due to their disability as captured by their RUGs score, as they are captured for all other SNF residents. Factors associated with higher facility inpatient costs included higher local wages (0.143, *P* < 0.001), greater number of admissions and inpatient days (0.030 for admissions and 0.643 for inpatient days, *P* < 0.001), and government ownership (0.119, *P* = 0.002).

## 4. Discussion

We investigated whether the reason why MS patients may be facing barriers to SNF care access because their care may be more costly than what facilities spend on other SNF residents. We found no significant association between the percentage of MS residents-days in an SNF in a given year and facility inpatient costs. We also found that government-owned SNFs were more likely to care for MS patients and that such facilities tended to have higher daily costs.

This finding could have several interpretations. First, government-owned facility may be less likely to discriminate against patients with unique needs and those who could potentially be a high burden on staff, like patients with MS. Second, these high-cost facilities are likely to have greater staff-to-residents ratio and may be able to provide more specialized services which patients with MS require and seek (e.g., mental health and psychological counseling; physical, occupational, and speech rehabilitation; and therapy [20]). As a result, larger facilities may attract and admit more MS patients. Finally, public/government-sponsored facilities may serve a greater proportion of Medicaid-eligible residents including MS patients who often become eligible for Medicaid because of disability-related unemployment, long history of high medical bills, and, subsequently, poverty. 

Our results also demonstrated that facilities with higher percentage of MS residents had lower resident turn-around and, actually, had lower CMI (average patient severity of illness). This is consistent with prior findings that MS residents are more likely to have permanent (custodial) SNF stays rather than transient stays, for rehabilitation or care-giver respite [21]. Earlier studies also have indicated that MS patients with both functional and cognitive impairment were more likely to have a permanent nursing home admission [2].

We also note that, on average, the percentage of MS patients among SNF residents (1.2%) was higher than that of MS prevalence among general population (0.1%) of the same age [22]. This supports earlier reports [2–4] that MS patients may have greater long-term and institutional care needs compared with general population. Our findings that SNFs in the South tended to have fewer residents with MS are consistent with the epidemiology of MS and the fact that MS prevalence is higher in the Northern regions compared with the Southern parts of the USA. 

The fact that our study did not demonstrate an association between MS and SNF costs may have several explanations. Using the data from a national survey of informal caregivers, Buchanan et al. (2010) [2] demonstrated that, in MS patients, age, bowel dysfunction, poorer health, functional decline, and caregiver burden were associated with increased probability of SNF admission. This is different from the general population where the need for long-term SNF care is often determined by patient cognitive status. MS patients are younger and have higher education and better cognitive status [23] but worse functional status than a typical SNF resident. Current RUG-III systems include multiple qualifiers that may help better match level of a resident's need with facility reimbursement including ADL scores, special care (MS, tube feeding, and pressure ulcers), and impaired cognition among others. It is conceivable that the protective effect of age and education (positive predictors for cognition) and the negative effect of functional deficiencies (negative predictor of cognitive decline) cancel each other in terms of SNF costs. Bowblis (2012) [24] also suggested that patients with long-term care needs and higher socioeconomic statuses may choose to reside in assisted-care facilities instead of SNFs. Similarly, Buchanan (2006) reported that the use of physical and occupational therapies by residents with MS at admission to the nursing facility was significantly associated with payment source, controlling for other independent variables [25]. They concluded that when reimbursement was available, these therapies were more likely to be prescribed suggesting that type of health insurance coverage would be associated with patient SNF expenses. Finally, MS patients with greater resources and informal support may be benefiting from nursing home transitions programs that became available since the late 1990s [26].

Because of the nature and timeframe of the administrative data used in this study, our analysis may have several limitations. First, the costs data were aggregated at the facility level, and, hence, we could only make indirect inferences about individual patient costs [27]. We could not assess whether the patients with MS who received SNF care were representative of the entire population of MS patients with SNF needs, nor could we confirm that MS patients who did receive SNF care were selectively admitted to SNF because their perceived needs (at the time of admission) were comparable with facility resources. Another limitation of the analysis presented here is the lack of information about quality of care MS patients receive in these facilities. It is conceivable that in order to keep the costs similar for patients with special healthcare needs (like residents with MS) and other SNF residents, SNF administrators limit either quantity or selection of services necessary for patients with MS [25]. Furthermore, behavioral and cognitive problems are common among long-term care residents and may substantially influence cost of their care [6]. However, this study did not take into account the absence of mental or behavioral health diagnoses or symptoms among facility residents with or without MS. Finally, one may speculate that since resource utilization groups- (RUGs-) based case-mix measurement system [12] was introduced to calculate nursing home reimbursement while adjusting for the resident's severity of illness and resource use, facilities no longer have financial disincentive for selectively avoiding patients with heavier or unique care needs, such as MS patients. Results of the CMS-funded Staff Time and Resource Intensity Verification (STRIVE) Projects provide SNF staff time use data that could help researchers examine the quality of nursing home care, including residents with and without MS [13, 28].

In summary, we found that providing SNF care for a greater proportion of MS patients has no significant effect on facility costs. There is some evidence indicating that MS patients are more likely to reside in larger, government-owned facilities, which are also more likely to serve Medicaid population and provide a greater variety of services that MS patients need and seek out. More research is needed to understand the needs and attitudes towards institutional care among community-dwelling individuals with MS and to identify optimal strategies for providing high-quality and cost-effective skilled nursing care to MS population. In addition to costs, future studies should examine quality of care and health outcomes in this population of MS patients.

## 5. Summary

The study examined whether the costs of caring for young disabled adults with multiple sclerosis exceed those for a typical elderly skilled nursing facility patient. 

Multiple sclerosis (MS) affects younger adults and leads to permanent disability. The study examined whether the institutional costs of caring for young disabled adults with multiple sclerosis exceed the cost of care for a typical elderly skilled nursing facility patient, and we found the following.

- MS patients were no more costly than typical patients in skilled nursing facilities.
- MS patients tend to cluster in larger government-owned facilities located in the West and the Midwest of the USA.

It is unclear, however, how the efforts to maintain same-for-all cost of care may impact the quality of services for MS patients.

## Figures and Tables

**Table:** (a)

SNF characteristics	Facility MS volume		*P*
	Low (<3%)	High (>3%)	
	Number (%)	Number (%)	
All	12,508 (92%)	1,148 (8%)	
Region			<0.0001
Northeast	2,385 (19%)	208 (18%)	
Midwest	3,695 (30%)	465 (41%)	
South	4,679 (37%)	146 (13%)	
West	1,749 (14%)	329 (28%)	
Type of ownership			<0.0001
For profit	9,564 (76%)	840 (73%)	
Not for profit	2,546 (20%)	229 (20%)	
Governmental	398 (4%)	79 (7%)	

**Table:** (b)

	Mean (std. dev.)	Mean (std. dev.)	*P*
Cost per day	217 (107)	213 (89)	0.110
Inpatient days	36,556 (21)	33,204 (21)	<0.0001
Admissions	239 (218)	197 (162)	<0.0001
Case-mix index	0.939 (0.10)	0.926 (0.08)	<0.0001

MS: multiple sclerosis; SNF: skilled nursing facility; *P*: *P* value; std. dev.: standard deviation.

MS volume is defined as the annual percentage of patients-days attributed to multiple sclerosis.

**Table 2:** Multivariate analysis: facility odds of having high MS volume (defined by pct MS days > 3%).

Effect	OR	95% Wald confidence limits		*P* value
Region				
Midwest versus Northeast	1.300	1.084	1.559	0.005
South versus Northeast	0.338	0.270	0.422	<.0001
West versus Northeast	2.133	1.750	2.600	<.0001
Type of ownership				
“Not for profit” versus “for profit”	0.909	0.770	1.072	0.255
Governmental versus “for profit”	1.904	1.452	2.497	<.0001
Mean cost per day, $100 s	0.994	0.931	1.062	0.857
Mean *N* of inpatient days, 100 Ks	0.887	0.586	1.343	0.571
Mean *N* of admissions, 10 s	0.988	0.983	0.993	<.0001
Mean case-mix index (CMI)	0.924	0.448	1.904	0.831

OR = odds ratio; c-statistic = 0.776.

**Table 3:** Factors contributing to variation in MS days among facilities with high MS volume.

Number of SNFs	1,148		
Number of observations	5,947		

Effect	Estimate	Standard error	*P* value

Intercept	5.522	0.669	<.0001
Region			
Midwest versus Northeast	−0.014	0.165	0.933
South versus Northeast	−0.298	0.159	0.061
West versus Northeast	0.361	0.179	0.044
Type of ownership			
“Not for profit” versus “for profit”	−0.009	0.089	0.924
Governmental versus “for profit”	0.030	0.222	0.893
Mean cost per day, in $100	0.118	0.069	0.088
Mean *N* of inpatient days, 100 K	0.471	0.373	0.207
Mean *N* of admissions, in tens	−0.007	0.005	0.158
Mean case-mix index (CMI)	−1.733	0.678	0.011

SNF: skilled nursing facility; see the appendix for performance of the mixed model.

**Table 4:** Effect of MS volume on SNF inpatient costs (Model 1).

Parameters	Estimate	RSE	*P* value
Intercept	2.747	0.119	<.0001
Facility percent of MS days	−0.001	0.001	0.26
Facility mean %MS days	0.002	0.003	0.435
Log of wage	0.143	0.026	<.0001
Log of total case-mix-adjusted inpatient days	0.643	0.019	<.0001
Log of *N* of admissions adjusted for case mix	0.030	0.003	<.0001
Facility mean of log of wages	0.997	0.045	<.0001
Facility mean of log of total inpatient days	0.288	0.024	<.0001
Facility mean of log of total *N* of admissions	−0.007	0.008	0.367
Competition	−0.010	0.019	0.609
Not for profit	0.033	0.021	0.105
Governmental	0.119	0.038	0.002
Interaction of competition and NFP	0.044	0.032	0.168

Interaction of competition and governmental	0.029	0.057	0.613

RSE: robust standard error; MS: multiple sclerosis.

Log of total case-mix-adjusted inpatient days.

Facility wage is the county/year mean of average SNF wages. Wages were adjusted for inflation.

NFP: not for profit.

Using facilities' REs and AR (1) structure of residuals errors.

See the appendix for performance of the mixed model.

## Appendix

### Controlling for the Correlation among Multiple Observations Provided by the Same Facility

Since there were multiple records per facility, we adjusted for correlation among the observations in each facility (within facility variation). To insure robustness of our estimation strategy, we estimated four distinct models.

In the first model (M1), we explicitly controlled for facility effect by including fixed effects so that the effects of the variables can be interpreted as “within” facility effects while facility characteristics are accounted for. We modified this M1 model by replacing fixed with random facility effects (Res) (M2) and included facility-level means of the facility characteristics (i.e., *X*, *W*, CMO, and %MS) so that the effect of these variables is still “within” facility effect, net of facility characteristics. The rest of facility characteristics were controlled by facility simple REs. Next, we improved the model by specifying the covariance of residual (in addition to REs) error terms as autoregressive order 1, to model temporal correlation (M3). In the final model (M4), we added facility characteristics such as competition and type of ownership. In models M2–M4, we used robust (empirical) standard errors to account for possible departures from the chosen covariance structure.
